# Tobacco dependence severity, cessation legacy and serial mediation in aging China

**DOI:** 10.18332/tid/221187

**Published:** 2026-05-22

**Authors:** Wenli Li, Meng Liu, Wenbin Du

**Affiliations:** 1 Research Institute of Social Development, Southwestern University of Finance and Economics, Chengdu, China; 2 Department of Sociology, School of Public Administration, Hohai University, Nanjing, China

**Keywords:** tobacco dependence, consumption intensity, FTND consumption subscale, cessation legacy, serial mediation

## Abstract

**INTRODUCTION:**

Most studies on smoking in China operationalize tobacco use as a binary exposure, obscuring clinically meaningful heterogeneity in dependence intensity. Despite China’s status as the world’s largest tobacco consumer, limited research has examined gradients in tobacco dependence severity, post-cessation health legacies, or the behavioral-psychological pathways linking nicotine entrenchment to chronic disease in older adults.

**METHODS:**

This secondary analysis drew on data from the 2020 China Health and Retirement Longitudinal Study (CHARLS). Tobacco dependence severity was classified: as mild (<10 cigarettes/day), moderate (10–20), or severe (>20) using a consumption-based proxy aligned with the Fagerström test for nicotine dependence (FTND) consumption subscale. Analytic samples included current smokers for dependence–chronic disease associations and serial mediation (n=3637) and former smokers for pre-cessation dependence analyses (n=1935). Multivariable logistic regression estimated direct associations. Serial mediation through reduced physical activity and elevated depressive symptoms to chronic disease was tested using path analysis with bias-corrected bootstrapping. All models adjusted for gender, age, education level, hukou type (household registration status), income, employment, and marital status.

**RESULTS:**

Compared with mild dependence, moderate current dependence was associated with higher odds of any chronic disease (AOR=1.22; 95% CI: 1.04–1.42), while severe dependence showed a positive but marginally significant association (AOR=1.24; 95% CI: 0.99–1.54). Among former smokers, moderate and severe pre-cessation dependence predicted elevated hypertension (AOR=1.56 and 1.81) and dyslipidemia (AOR=1.77 and 1.89). Associations appeared more pronounced among agricultural hukou holders, though formal interaction tests were non-significant. Serial mediation identified a statistically significant but substantively trivial indirect pathway through reduced physical activity and elevated depressive symptoms (β=0.001, p<0.05); single-mediator paths were non-significant.

**CONCLUSIONS:**

Tobacco dependence severity, as measured by a cigarette consumption–based proxy, shows an overall positive gradient with chronic disease risk among older Chinese adults. Higher pre-cessation dependence is associated with persistent hypertension and dyslipidemia risk after quitting. Descriptive subgroup variation appeared more pronounced among agricultural hukou holders, although interaction tests did not support statistically significant heterogeneity. The findings suggest the value of moving beyond binary smoking classifications toward dependence-stratified screening and post-cessation surveillance. Such considerations may inform tobacco control efforts under Healthy China 2030 for rural populations.

## INTRODUCTION

Tobacco use remains the leading preventable cause of morbidity and premature mortality worldwide, responsible for approximately 8 million deaths annually and a disproportionate share of the global burden of chronic non-communicable diseases (NCDs), including cardiovascular disease, type 2 diabetes, cancer, and chronic obstructive pulmonary disease^1,2^. In China, the world’s largest tobacco consumer, population aging and persistent health inequalities have intensified concern about the long-term consequences of smoking in later life.

Despite this urgency, Chinese tobacco epidemiology has largely relied on a binary smoker versus non-smoker framework that obscures meaningful heterogeneity in the intensity and physiological entrenchment of nicotine dependence^3,4^. In high-income countries, prospective cohort studies have shown that smoking-attributable mortality and disease risk escalate steeply and non-linearly with daily cigarette consumption, a gradient masked by binary classifications^5,6^. The Fagerström test for nicotine dependence (FTND) provides an international standard for dependence assessment, with daily cigarette consumption serving as its primary subscale^7,8^. Whether a consumption-based proxy for tobacco dependence severity predicts chronic disease burden in aging Chinese populations remains empirically unexamined.

A second gap concerns the chronic disease consequences of prior dependence among individuals who have already quit. Cessation is universally endorsed as the most effective tobacco control strategy^9,10^, yet former smokers are routinely treated as a homogeneous category, obscuring the possibility that those more heavily dependent before quitting carry substantially different residual risk profiles. High-intensity prior nicotine exposure can induce persistent epigenetic changes, structural arterial remodeling, and accelerated telomere attrition that continue to elevate cardiometabolic risk years after cessation^11-13^. Whether pre-cessation dependence leaves a measurable ‘cessation legacy’ in hypertension and dyslipidemia among aging Chinese former smokers, has not been systematically examined.

Third, the behavioral and psychological pathways through which tobacco dependence intensity links to chronic disease remain underexplored. Higher dependence is associated with lower physical activity and elevated depressive symptoms, both established NCD risk factors^14,15^. These may operate sequentially: dependence erodes self-regulatory capacity for physical activity^16^, which in turn removes a key buffer against depression, allowing depressive affect to accumulate through neuro-immunological pathways that promote disease^17,18^. The Bandura^16^ self-efficacy theory provides the mechanistic bridge for this ordered sequence.

To integrate these observations, we draw on three complementary theoretical frameworks. Fundamental Cause Theory posits that socio-economic and structural factors^19^, such as China’s hukou (household registration) system, shape access to health-promoting resources and exposure to risks, thereby conditioning the health consequences of any given behavioral factor. Health Lifestyle Theory suggests that tobacco dependence severity constrains the behavioral repertoire available to smokers^20^, making health-promoting activities like regular physical activity less likely. The Stress Process Model further proposes that chronic stressors, including nicotine dependence, elevate psychological distress (depressive symptoms), which in turn increases disease risk through neuro-endocrine and inflammatory mechanisms. Taken together, these frameworks imply a serial pathway in which structural position (hukou) moderates the cascade from tobacco dependence to reduced physical activity, then to elevated depressive symptoms, and ultimately to chronic disease. This integrated perspective also highlights that former smokers with higher pre-cessation dependence may carry a persistent health legacy, as prior entrenchment leaves lasting biological and behavioral imprints even after quitting.

This study addresses these gaps using nationally representative data from the 2020 China Health and Retirement Longitudinal Study (CHARLS). Specifically, the analysis examines whether a cigarette consumption-based proxy for tobacco dependence severity is associated with chronic disease in a graded fashion among current smokers; whether pre-cessation levels in this proxy are associated with persistent hypertension and dyslipidemia risk among former smokers; and whether physical activity and depression sequentially mediate the association.

## METHODS

### Study design and data sources

This study is a secondary analysis of data from the 2020 wave of the China Health and Retirement Longitudinal Study (CHARLS). CHARLS is a nationally representative survey of Chinese adults aged ≥45 years and their households, jointly administered by Wuhan University and Peking University. It uses a stratified, multistage probability sampling design and covers more than 10000 households across 150 counties/districts and 450 village-level units. The national baseline survey was conducted in 2011, with follow-up waves in 2013, 2015, 2018, and 2020. The 2020 survey includes detailed information on respondents’ health conditions and health-related behaviors, health insurance participation and utilization, family structure and intergenerational support, socio-economic status, and COVID-19-related topics.

The present study draws on the 2020 individual-level CHARLS data and links respondents to household- and community-level information using unique identifiers. Eligible participants were respondents in the 2020 wave who completed the key survey modules and had non-missing information on the dependent variable, focal independent variable, mediators, and covariates. Missing data were handled using a uniform listwise-deletion rule, such that any observation with missing information on a key study variable was excluded from the analysis. The resulting analytic sample sizes are reported in Table 1. Definitions, measurements, and coding schemes for all key variables are summarized in Supplementary file Table 1.

**Table 1:** Descriptive statistics for all variables, secondary analysis of the CHARLS 2020

*Variables*	*Mean*	*SD*	*Total* *n*
**Age** (years)	62.94	9.28	12269
**Years of education**	5.87	4.27	12269
**Log total income**	8.81	1.62	12269
**Depression symptoms** (CES-D score)	18.26	6.36	12269
	**n**	**%**	
**Presence of chronic disease** (ref. no)	7330	37.85	19367
**Current level of tobacco dependence** (ref. mild)			
Mild	1929	39.28	4911
Moderate	2291	46.65	
Severe	691	14.07	
**Pre-cessation level of tobacco dependence** (ref. mild)			
Mild	808	33.09	2442
Moderate	983	40.25	
Severe	651	26.66	
**Current smoking status** (ref. still smoke)			
Still smoke	4911	60.82	8075
Quit	2565	31.76	
Never smoked	599	7.42	
**Physical activity** (ref. no)	8467	49.36	17152
**Gender** (ref. male)	10287	53.12	19367
**Marital status** (ref. other)			
Married with spouse present	14578	75.27	19367
**Hukou** (ref. agricultural)			
Agricultural	14416	74.48	19356
Non-agricultural	2904	15.00	
Unified residence	2036	10.52	
**Work status** (ref. no)	5808	30.02	19344

The original CHARLS data collection was approved by the appropriate institutional ethics review bodies, and written informed consent was obtained from all participants before participation. The present study uses only de-identified data, and the authors had no access to any information that could identify individual participants. The study involved no direct contact with participants and no intervention. In accordance with institutional policy for secondary analyses of publicly available de-identified data, no additional ethics review was required for this study.

### Variables

*Independent variables*

Tobacco dependence is the independent variable. Leveraging the measurement of smoking behaviors available in CHARLS, this work operationalizes tobacco dependence following the logic of the Fagerström test for nicotine dependence (FTND)^21^. The FTND is a standardized instrument designed to assess nicotine dependence among adult smokers and comprises six items capturing core dimensions of dependence, including time to first cigarette after waking, cigarettes smoked per day, and difficulty refraining from smoking in restricted settings.

Among the datasets available for this study, including the China Family Panel Studies (CFPS), the China Health and Retirement Longitudinal Study (CHARLS), and the Chinese General Social Survey (CGSS), CHARLS provides the most detailed information on smoking behavior. However, its smoking-related items do not support a full operationalization of tobacco dependence in accordance with the Fagerström test for nicotine dependence (FTND). Tobacco dependence was therefore not measured using the complete FTND scale. Instead, cigarettes smoked per day were used as a consumption-based proxy for tobacco dependence severity. Given that cigarette consumption constitutes a central component of the FTND framework, the CHARLS item, ‘On average, how many cigarettes do you smoke per day?’, was used to classify respondents into three levels: 0–10 (mild), 11–20 (moderate), and ≥21 cigarettes/day (severe).

This classification logic is derived from the cigarettes-per-day item in the FTND framework and has been widely used in social science and population health research^22^, supporting both its comparability and substantive interpretability. Accordingly, these categories should be understood as smoking-intensity-based approximations of dependence severity rather than formal FTND classifications.

Current smoking status is measured as a three-category variable distinguishing current smokers, former smokers, and never smokers. In the empirical analyses, smoking status is entered as a set of indicator variables, with current smokers serving as the reference group.

*Dependent variables*

The primary outcome is a binary indicator of whether the respondent had been diagnosed with any chronic disease. This composite measure is used to capture overall morbidity burden in later life, given the high prevalence of co-occurring chronic conditions in aging populations. Chronic disease status is measured based on respondents’ reports of physician-diagnosed conditions and summarized as the total number of chronic conditions across 15 items. Each condition is coded as present or absent, with non-substantive responses treated as missing. The resulting count ranges from 0 to 15, with higher values indicating greater comorbidity burden. Respondents missing on all condition items are coded as missing on the count measure. Building on this count, a dichotomous outcome, any chronic disease, is constructed to indicate whether at least one chronic condition is reported. Respondents reporting one or more physician-diagnosed conditions are coded as having any chronic disease, whereas those reporting none are coded as not having any chronic disease. Cases missing on all condition items are treated as missing on this binary measure and excluded from the analytic sample.

In addition, six selected tobacco-related conditions are retained for disease-specific analyses: hypertension, dyslipidemia, diabetes or elevated blood glucose, chronic lung disease, heart disease, and stroke. Each condition is coded as a binary indicator of physician diagnosis, with non-substantive responses treated as missing. This coding scheme promotes comparability across the selected disease-specific measures. The original questionnaire items, definitions, coding schemes, and operationalization procedures for all variables included in the analysis are reported in Supplementary file Table 1.

*Mediating variables*

Physical exercise participation is measured as a binary indicator based on whether the respondent reports engaging in any activity for the purpose of physical exercise. Activities of vigorous, moderate, and light intensity are considered. Respondents are classified as participating in physical exercise if at least one reported activity is undertaken for exercise, and as not participating otherwise. Cases with insufficient information on all relevant activity items are treated as missing and excluded from subsequent analyses. This measure, therefore, captures participation in physical exercise rather than the frequency of exercise.

Depressive symptoms are measured using a 10-item scale that captures how frequently respondents experienced specific feelings or behaviors during the past week. Two positively worded items are reverse-coded so that higher values consistently indicate greater depressive symptom severity. Item scores are then summed to construct a continuous depressive symptom score, with higher values indicating more severe depressive symptoms. Non-substantive responses are treated as missing, and a consistent missing-data rule is applied when computing the total score.

*Control variables*

Covariates include gender, age, marital status, years of schooling, hukou type, income, and employment status. Gender is measured as a binary variable, and age as a continuous variable in years. Both variables are derived from harmonized CHARLS baseline information to ensure consistency across respondents. Marital status and hukou type are included as categorical covariates. Cases reporting no hukou are treated as missing.

Education level is measured using a harmonized education variable that combines current and prior-wave information, where available, to improve consistency across respondents. It is recoded into years of schooling and included in the models as a continuous variable, with higher values indicating more years of education. Income is measured as the sum of annual wage income and annual transfer income. This combined measure is used to capture respondents’ total annual income and is log-transformed for analysis. Higher values indicate higher total income, and cases with invalid or insufficient information are treated as missing. Employment status is measured as a binary variable indicating whether the respondent is currently employed or attached to a job at the time of the interview. Respondents with insufficient information to determine employment status are treated as missing.

### Statistical analysis

Descriptive analyses were first conducted to summarize all study variables (Table 1). Continuous variables are presented as means and standard deviations, and categorical variables as frequencies and percentages. Bivariate correlations among the main variables were also examined. Second, because the outcome variable – whether the respondent had any chronic disease – was dichotomous, binary logistic regression models were estimated to assess the association between tobacco dependence and chronic disease risk. Tobacco dependence was entered as a set of categorical indicators with an explicit reference group to avoid imposing a linear assumption on an ordinal measure. Covariates included gender, age, years of schooling, hukou type, income, current employment status, and marital status. For ease of interpretation, regression results are presented as adjusted odds ratios (AORs) with 95% confidence intervals (CIs).

Third, a serial mediation path model was then estimated to assess whether tobacco dependence was associated with chronic disease risk through physical activity and depressive symptoms. Because the outcome variable and the first mediator were dichotomous, and the focal predictor was an ordinal categorical measure, the mediation model was estimated in Mplus 8.3 using weighted least squares mean and variance adjusted (WLSMV) estimation with a probit link for endogenous categorical variables^23^. Indirect effects were evaluated using bias-corrected bootstrap confidence intervals based on 5000 resamples^24^, and model fit was assessed using multiple indices, including CFI, TLI, RMSEA, and SRMR^25^.

Finally, the serial mediation model was estimated in the pooled sample to capture the overall pathway linking tobacco dependence, physical activity, depressive symptoms, and chronic disease. This strategy was guided by the interaction analyses in the main models, which did not provide robust evidence of heterogeneity by hukou status or sex. In addition, subgroup-specific mediation models were likely to suffer from limited statistical power and unstable estimates, particularly among women. As a sensitivity analysis, an additional serial mediation model was estimated in the male subsample. Unless otherwise noted, statistical significance was defined as p<0.05. Descriptive statistics and regression analyses were conducted in Stata 19, and the mediation models were estimated in Mplus 8.3.

## RESULTS

### Descriptive statistics

Table 1 summarizes the descriptive characteristics of the analytic sample. Respondents had a mean age of 62.94 years and 5.87 years of schooling on average. The mean log total income was 8.81, and the mean depressive symptoms score was 18.26. Overall, 37.85% of respondents reported at least one chronic disease. Among current smokers, moderate tobacco dependence was the most prevalent category (46.65%), followed by mild (39.28%) and severe dependence (14.07%). A similar distribution was observed for pre-cessation dependence among former smokers, although the proportion with severe dependence was higher (26.66%). In the full sample, 60.82% were current smokers, 31.76% were former smokers, and 7.42% had never smoked; 49.36% reported engaging in physical activity. The sample was predominantly female (53.12%), married and living with a spouse (75.27%), and agricultural hukou holders (74.48%).

### Logistic regression

Model 1 in Table 2 estimates the association between current tobacco dependence and chronic disease while adjusting for the study covariates. With mild dependence as the reference category, higher dependence is associated with significantly greater odds of chronic disease: moderate dependence is linked to a 21.5% increase in the odds (AOR=1.22; 95% CI: 1.04–1.42), and severe dependence to a 23.6% increase (AOR=1.24; 95% CI:0.99–1.54). These findings suggest that net of sociodemographic characteristics, chronic disease risk rises with the intensity of tobacco dependence, with the association modestly stronger at higher dependence levels. Turning to covariates, women have significantly higher odds of chronic disease than men (AOR=1.27; 95% CI:0.97–1.66). Age is positively associated with chronic disease (AOR=1.01; 95% CI: 1.00–1.02), indicating that each additional year is associated with an approximately 1.4% increase in the odds. Relative to respondents with agricultural hukou, those with non-agricultural hukou exhibit significantly higher odds of chronic disease (AOR=1.34; 95% CI: 1.08–1.66; 33.8% higher). By contrast, employed respondents have significantly lower odds than those not working (AOR=0.77; 95% CI: 0.64–0.92; 23.3% lower).

**Table 2:** Logistic regression results of the direct effects of tobacco dependence on chronic disease risk, CHARLS 2020

*Variables*	*Model 1* *AOR (95% CI)*	*Model 2* *AOR (95% CI)*	*Model 3* *AOR (95% CI)*
**Total,** n	3637	1935	6114
**Age**	1.01*** (1.00–1.02)	1.02** (1.00–1.03)	1.01*** (1.00–1.09)
**Years of education**	1.01 (0.99–1.04)	1.01 (0.98–1.04)	1.01* (1.00–1.03)
**Log total income**	1.02 (0.97–1.08)	0.94 (0.87–1.02)	0.99 (0.95–1.04)
**Current level of tobacco dependence** (ref. mild)			
Moderate	1.22** (1.04–1.42)		
Severe	1.24* (0.99–1.54)		
**Gender** (ref. male)	1.27* (0.97–1.66)	1.27 (0.89–1.83)	1.26** (1.04–1.52)
**Marital status** (ref. other)	0.91 (0.77–1.07)	1.01 (0.80–1.27)	0.93 (0.82–1.05)
**Hukou status** (ref. agricultural)			
Non-agricultural	1.34*** (1.08–1.66)	1.08 (0.81–1.43)	1.19** (1.02–1.40)
Unified residence	1.07 (0.84–1.36)	1.22 (0.88–1.69)	1.13 (0.94–1.36)
**Work status** (ref. no)	0.77*** (0.64–0.92)	0.83 (0.65–1.07)	0.79*** (0.69–0.92
**Pre-cessation level of tobacco dependence** (ref. mild)			
Moderate		1.09 (0.88–1.35)	
Severe		1.16 (0.91–1.49)	
**Current smoking status** (ref. still smoke)			
Quit			1.42**** (1.27–1.59)
Never smoked			1.19* (0.97–1.46)
**Constant**	0.17**** (0.07–0.40)	0.45 (0.14–1.39)	0.27**** (0.14–0.52)

AOR: adjusted odds ratio. Model 1 includes current tobacco dependence and the control variables. Model 2 includes pre-cessation level of tobacco dependence and the control variables. Model 3 includes current smoking status and the control variable.

**** p<0.001,

*** p<0.01,

** p<0.05,

* p<0.1.

Model 2 examines the association between pre-cessation tobacco dependence and chronic disease risk with the same set of controls. Relative to mild pre-cessation dependence, neither moderate (AOR=1.09; 95% CI: 0.88–1.35) nor severe dependence (AOR=1.16; 95% CI: 0.91–1.49) is statistically significant at conventional thresholds. This pattern indicates that, within the former-smoker subsample, dependence classifications based solely on pre-cessation cigarettes per day have limited predictive value for current chronic disease risk. Among the covariates, age is the only factor that remains positively and significantly associated with chronic disease (AOR=1.02; 95% CI: 1.00–1.03).

Model 3 further assesses the association between current smoking status and chronic disease, also net of the study covariates. With current smokers as the reference group, former smokers have significantly higher odds of chronic disease (AOR=1.42; 95% CI:1.27–1.59). Never smokers also exhibit higher odds than current smokers, although this contrast attains statistical significance only under a more lenient 90% criterion. Taken together, the elevated risk observed among former smokers is more consistent with health selection and reverse causality: chronic disease diagnosis, symptom onset, or clinical advice may induce smoking cessation, generating the cross-sectional pattern in which former smokers appear to be at higher risk. In parallel, the current-smoker group may be shaped by selective survival and health-based selection.

Taken together, the results in Table 2 indicate that current tobacco dependence is consistently and positively associated with chronic disease risk (Model 1). In contrast, within the former-smoker subsample, the findings indicate no statistically significant gradient by pre-cessation dependence (Model 2). Model 3 further shows that smoking status is significantly associated with chronic disease, with former smokers facing a higher risk than current smokers.

Supplementary file Table 2 evaluates within the former-smoker subsample whether pre-cessation tobacco dependence is associated with two chronic disease outcomes. Using mild pre-cessation dependence as the reference category, pre-cessation dependence is positively and significantly related to both hypertension and dyslipidemia, with an overall positive gradient. In the hypertension model (Model 1), the adjusted odds ratios (AOR) for moderate and severe dependence are 1.56 (95% CI: 1.02–2.37) and 1.81 (95% CI: 1.14–2.87), respectively (both p<0.05). In the dyslipidemia model, the corresponding odds ratios are 1.77 (95% CI: 1.17–2.67) and 1.89 (95% CI: 1.19–3.00) (both p<0.01). Taken together, higher levels of pre-cessation dependence are associated with elevated chronic disease risk in a pattern consistent with cumulative exposure.

Supplementary file Table 3 compares representative smoking-related chronic disease risk across smoking-status groups net of covariates, using current smokers as the reference category. Disease-specific analyses were restricted to conditions with relatively well-established links to tobacco exposure in the epidemiological and public health literature.

Former smokers exhibit higher odds across all eight chronic conditions examined, with most estimates statistically significant: hypertension (AOR=1.29; 95% CI: 1.04–1.59), dyslipidemia (AOR=1.27; 95% CI: 1.04–1.57), diabetes (AOR=1.40; 95% CI: 1.09–1.82), chronic lung disease (AOR=1.37; 95%CI: 1.07–1.75), heart disease (AOR=1.68; 95% CI: 1.32–2.15), and stroke (AOR=1.59; 95% CI: 1.10–2.30).

Accordingly, Supplementary file Table 3 is best interpreted as describing the correlational structure between smoking status and chronic disease prevalence rather than the causal impact of smoking cessation on health.

Collectively, Supplementary file Tables 2 and 3 point to two complementary patterns. Supplementary file Table 2, estimated within the former-smoker subsample, documents a clear gradient in prior exposure, underscoring that former smokers are not a homogeneous group: chronic disease risk varies meaningfully with pre-cessation dependence intensity and cumulative smoking exposure. Supplementary file Table 3, which contrasts current smoking-status groups, reveals a consistent cross-disease association whereby former smokers, relative to current smokers, exhibit higher risk across six chronic disease outcomes, with most estimates statistically significant. These findings are not contradictory. The former emphasizes within-former-smoker heterogeneity in accumulated exposure, whereas the latter reflects the potentially selective linkage between smoking cessation and subsequent health shocks.

### Heterogeneity and interaction analyses

Heterogeneity was assessed by estimating subgroup-specific models and by formally testing interaction terms between tobacco dependence and hukou status, as well as between tobacco dependence and sex (Supplementary file Table 4). Given the potential underreporting of smoking among women in China, an additional men-only specification was estimated as a robustness check.

Formal interaction tests did not provide evidence of statistically significant heterogeneity by hukou status. The interaction terms between tobacco dependence and non-agricultural hukou were not significant for either moderate dependence (AOR=0.87; 95% CI: 0.63–1.21) or severe dependence (AOR=0.98; 95% CI: 0.60–1.60). Likewise, formal interaction tests did not support statistically significant heterogeneity by sex. The interaction term for moderate dependence among women was only marginal (AOR=1.59; 95% CI: 0.91–2.78), whereas the interaction term for severe dependence was null (AOR=1.00; 95% CI: 0.26–3.78).

Consistent with the non-significant interaction terms, the subgroup-specific estimates in Supplementary file Table 5 are better interpreted as descriptive variation rather than as evidence of robust heterogeneity. In the male subsample, both moderate and severe dependence were positively associated with chronic disease risk (AOR=1.17; 95% CI: 1.00–1.38; AOR=1.22; 95% CI: 0.97–1.53, respectively), indicating a relatively stable pattern. In the female subsample, the coefficient for moderate dependence was larger (AOR=2.06; 95% CI: 1.19–0.58), whereas the estimate for severe dependence was not statistically significant (AOR=1.30; 95% CI: 0.34–4.92), suggesting a less stable within-group pattern. Across hukou groups, the estimates were descriptively stronger among agricultural-hukou holders (AOR=1.28; 95% CI: 1.07–1.54 for moderate dependence; AOR=1.27; 95% CI: 0.98–1.64 for severe dependence) than among respondents with non-agricultural and unified hukou (AOR=1.09; 95% CI: 0.82–1.44 and AOR=1.17; 95% CI: 0.77–1.80, respectively). These subgroup differences should therefore be interpreted cautiously, especially given the lack of statistically significant interaction effects.

Given the likely underreporting of smoking among women in China, a men-only analysis was emphasized as a robustness check. In the male subsample (Supplementary file Table 5), current tobacco dependence remained positively associated with chronic disease, with AOR of 1.17 for moderate dependence and 1.22 for severe dependence.

Smoking among women in China is likely to be underreported because of strong gendered social norms and social desirability pressures. This may attenuate the observed associations in the pooled sample, reduce the reliability of female-specific estimates, and limit the interpretability of sex-based subgroup comparisons. The men-only analyses are therefore particularly informative as a robustness check.

### Serial mediation analysis

Supplementary file Table 6 reports Pearson correlations among variables used in the path analysis (n=3637). Overall, bivariate associations among the focal constructs are modest. Tobacco dependence is not significantly correlated with chronic disease (r=0.0108), but is significantly and inversely related to physical activity (r= -0.1076, p<0.001), indicating lower activity levels among more dependent respondents. Chronic disease is positively and more strongly correlated with depressive symptoms (r= 0.1341, p<0.001), whereas physical activity is negatively correlated with depressive symptoms (r= -0.0290, p<0.001). This pattern is consistent with a relatively robust psychosomatic comorbidity linkage, while the activity-mental health association, though statistically detectable, is substantively small. Notably, chronic disease also exhibits a small but significant positive correlation with physical activity (r=0.0407, p<0.001).

Covariates-including gender, age, education level, hukou, income, and employment status, are also correlated with the focal variables to varying degrees. For instance, age is positively correlated with chronic disease, physical activity, and depressive symptoms, but negatively correlated with tobacco dependence; education level is strongly positively correlated with income and inversely correlated with depressive symptoms; and employment status is positively correlated with tobacco dependence but negatively correlated with chronic disease and depressive symptoms (Supplementary file Table 6).

Two correlations run counter to conventional expectations. Tobacco dependence is weakly negatively correlated with depressive symptoms (r= -0.0426, p<0.01), and chronic disease is weakly positively correlated with physical activity (r=0.0407, p<0.001). These ‘counterintuitive’ directions are more plausibly attributable to selective health behaviors and reverse causality in cross-sectional data. Individuals with chronic conditions may be more likely to initiate or report physical activity in response to medical advice or self-management, producing the appearance that those with disease exercise more. Likewise, the negative dependence-depression correlation may reflect sample heterogeneity and unobserved confounding (e.g. occupational or social roles, health selection, and coping strategies), underscoring that bivariate correlations should not be interpreted as net or causal effects.

Using Mplus 8.3^23^, this work estimates a serial mediation path model to examine whether the current level of tobacco dependence (IV) is associated with chronic disease risk (DV) through the sequential pathway from physical activity (M1) to depressive symptoms (M2). Physical activity (exercise participation) and chronic disease (any chronic disease) are modeled as binary outcomes, whereas depressive symptoms are treated as a continuous measure. Because the model includes endogenous dichotomous variables, this work estimates parameters using the WLSMV estimator for categorical outcomes and specifies a probit link under DELTA parameterization, yielding robust coefficients and standard errors. All models adjust for gender, age, marital status, years of schooling, hukou type, log of household income, and employment status. Missing values are coded as -999 and treated as system missing. The analytic sample for estimation comprises (n=3637) respondents. Model fit is generally acceptable [χ^2^ (23)=10.29, p=0.01; CFI=0.97, RMSEA= 0.02, and SRMR=0.018]. Because the χ^2^ statistic is well known to be sensitive to trivial deviations from exact fit in large samples, especially with relatively few degrees of freedom, this study places greater emphasis on RMSEA, CFI, and SRMR, which collectively indicate good model fit ^25^ (Table 3).

**Table 3:** Structural path coefficients from the serial mediation path model, CHARLS 2020 (N=3637)

*Variable*	*Predictor*	*Path*	*Std. Estimate*	*95% CI*
M1	IV	a1	-0.081***	-0.12 – -0.04
M2	M1	d21	-0.051*	-0.10 – -0.00
M2	IV	a2	-0.017	-0.05–0.02
DV	M2	b2	0.173***	0.13–0.22
DV	M1	b1	0.062*	0.00–0.12
DV	IV	c	0.058**	0.02–0.10

IV: independent variable (current tobacco dependence). M1: first mediator (physical activity). M2: second mediator (depressive symptoms). DV: dependent variable (chronic disease risk). This table reports completely standardized (STDYX) coefficients and 95% confidence intervals from the serial mediation path model. The model is estimated using WLSMV with a probit link; estimates for control variables are omitted for brevity.

*** p<0.001,

** p<0.01,

* p<0.05.

**Figure 1:**
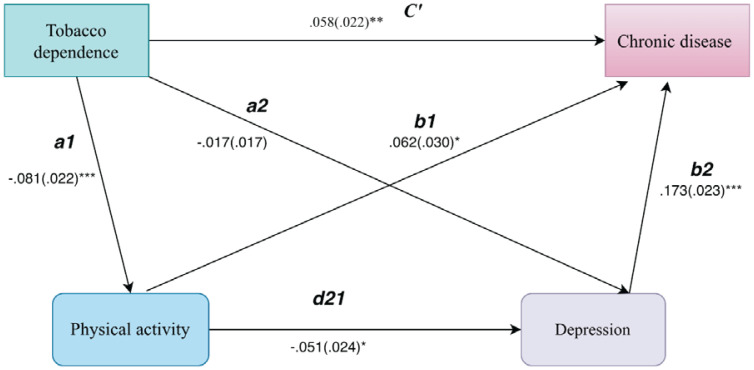
Path diagram of the serial mediation model, CHARLS 2020 (N=3637)

Net of covariates, including gender, age, marital status, years of schooling, hukou type, income, and employment status, the serial mediation model in Table 4 indicates an overall positive association between tobacco dependence and chronic disease risk (total effect, STDYX=0.051; 95% CI: 0.01–0.09; p<0.05). After adding physical exercise (M1) and depressive symptoms (M2), the direct effect of tobacco dependence remains statistically significant (c'=0.058; 95% CI: 0.02–0.10; p<0.01), indicating that the association is driven primarily by a direct pathway rather than being fully conveyed through the mediators. The path estimates further indicate that tobacco dependence is associated with a lower likelihood of engaging in physical exercise (path a1 in Table 3: STDYX= -0.081; 95% CI: -0.12 – -0.04; p<0.001). Physical exercise, in turn, is inversely associated with depressive symptoms (path d21 in Table 3: STDYX= -0.051; 95% CI: -0.10 – -0.00; p<0.05), and depressive symptoms are strongly and positively related to chronic disease risk (path b2 in Table 3: STDYX=0.173; 95% CI: 0.13–0.22; p<0.001). Taken together, the direction of these coefficients aligns with the hypothesized chain linking tobacco dependence to chronic disease through reduced physical activity and worse mental health: higher dependence is associated with lower engagement in health-promoting behavior and elevated depressive symptoms, which in turn correspond to greater chronic disease risk (Figure 1).

**Table 4:** Mediation analysis: tobacco dependence and chronic disease, CHARLS 2020 (N=3637)

*Effect type*	*Pathway*	*Std. Estimate (STDYX)*	*95% CI*
Total effect	IV → DV	0.051*	0.01–0.09
Direct effect (c ')	IV → DV	0.058**	0.02–0.10
Total indirect effect	Σ indirect	-0.007	-0.02–0.00
Specific indirect 1	IV → M1 → DV	-0.005	-0.01–0.001
Specific indirect 2	IV → M2→ DV	-0.003	-0.01–0.00
Specific indirect 3 (serial)	IV → M1→ M2→ DV	0.001*	0.00–0.00

IV: independent variable (current tobacco dependence). M1: first mediator (physical activity). M2: second mediator (depressive symptoms). DV: dependent variable (chronic disease risk). This table reports completely standardized (STDYX) coefficients and 95% confidence intervals from the serial mediation path model. The model is estimated using WLSMV with a probit link; estimates for control variables are omitted for brevity.

*** p<0.001,

** p<0.01,

* p<0.05.

However, the mediation decomposition in Table 4 indicates that the overall indirect effect is small and not statistically significant (Σ indirect= -0.007). The specific indirect effects are similarly modest: neither the single-mediator pathway via physical exercise (-0.005) nor the single-mediator pathway via depressive symptoms (-0.003) reaches conventional significance levels. Only the serial indirect effect is positive and statistically significant (0.001, p<0.05). Thus, net of covariates, the data provide evidence of a detectable but substantively very small sequential pathway linking tobacco dependence to chronic disease risk through reduced exercise and elevated depressive symptoms.

Several implications follow. First, the pattern statistically significant component paths alongside a limited product term suggest that physical exercise and depressive symptoms likely operate as a secondary mechanism rather than the primary channel linking tobacco dependence to chronic disease. Second, the positive sign of the serial indirect effect is theoretically consistent with the estimated path directions (Table 3): tobacco dependence is negatively associated with physical exercise (greater dependence, less exercise); physical exercise is negatively associated with depressive symptoms (more exercise, fewer symptoms); and depressive symptoms are positively associated with chronic disease risk (more symptoms, higher risk). The implied indirect effect is therefore positive, indicating that increases in tobacco dependence can raise chronic disease risk through reduced exercise, elevated depressive symptoms, and, in turn, greater disease risk (Figure 1). This direction accords with health-lifestyle and stress-process perspectives, in which tobacco dependence constitutes not only a behavioral risk factor but also a condition that can erode health-promoting investments and undermine psychological well-being, thereby accumulating into chronic disease risk.

Finally, the ‘weak significance’ of the serial pathway should be interpreted in light of the full indirect-effect profile. Table 4 shows a negative but nonsignificant total indirect effect (-0.007), alongside negative (and nonsignificant) single-mediator indirect effects through M1 (-0.005) and M2 (-0.003), yet a positive and significant serial component (0.001). This configuration implies offsetting indirect processes: the positive serial pathway is counterbalanced by larger magnitude negative single-mediator components, yielding an overall indirect effect that is negative and statistically indistinguishable from zero. More broadly, the results suggest that the tobacco dependence-chronic disease association is unlikely to operate through a single mechanism; instead, multiple pathways may coexist and partially countervail one another at the aggregate level.

Notably, Table 3 also indicates a positive direct association between physical exercise and chronic disease (path b1: STDYX=0.062; 95% CI: 0.00–0.12; p<0.05), which contrasts with the expected protective effect of exercise. This counterintuitive pattern likely reflects, first, measurement limitations of the binary exercise indicator, which cannot differentiate intensity, frequency, or underlying purpose, and second, selection and reverse-causality processes whereby individuals with chronic conditions may be more likely to initiate or report exercise in response to clinical advice or self-management needs. Accordingly, the mediation results should be interpreted with caution. Future work would benefit from more granular measures of exercise frequency and intensity and from stronger adjustment for baseline health and functional status to assess the robustness of the proposed serial pathway.

## DISCUSSION

The analysis of the 2020 China Health and Retirement Longitudinal Study reveals an overall positive gradient between a cigarette consumption-based proxy for tobacco dependence severity and chronic disease prevalence among older Chinese adults. Compared with mild dependence, moderate current dependence was associated with higher odds of any chronic disease (AOR=1.22; 95% CI: 1.04–1.42), while severe dependence showed a positive but marginally significant association (AOR=1.24; 95% CI: 0.99–1.54). This broadly graded pattern indicates that binary smoking classifications obscure variation in dependence intensity.

Among former smokers, higher pre-cessation dependence was associated with elevated hypertension (moderate AOR=1.56; 95% CI: 1.02–2.37; severe AOR=1.81; 95% CI: 1.14–2.87) and dyslipidemia (moderate AOR=1.77; 95% CI: 1.17–2.67; severe AOR=1.89; 95% CI: 1.19–3.00). These results suggest a biological cessation legacy that may persist after quitting. Former smokers also exhibited higher chronic disease prevalence than current smokers overall, a pattern consistent with health-selective cessation rather than a protective effect of quitting.

Serial mediation analysis identified a statistically significant but substantively trivial indirect pathway from dependence through reduced physical activity and elevated depressive symptoms to chronic disease (β=0.001, p<0.05), while single-mediator paths were non-significant. This small sequential association is consistent with self-efficacy mechanisms and the Stress Process Model^16,17^. Direct biological pathways likely predominate. Descriptive subgroup variation appeared more pronounced among agricultural hukou holders, although interaction tests did not support statistically significant heterogeneity. Sex-specific estimates should be interpreted cautiously, and men-only analyses were used as a robustness check.

Our findings indicate that cessation does not necessarily eliminate the health consequences of prior smoking. More intensive dependence may leave lasting effects through long-term cumulative damage and metabolic and cardiovascular pathways, yielding a post-cessation ‘health legacy’ reflected in higher disease risk. Importantly, the estimates capture heterogeneity within former smokers by prior dependence intensity; interpretation should therefore account for variation in cessation timing and post-cessation behavioral change. Future work could incorporate years since quitting and duration of prior exposure to assess whether these factors condition the observed gradient.

Our analyses also indicate that the elevated risk among former smokers is broadly consistent across outcomes and is especially pronounced for cardiometabolic and cerebrovascular conditions. In contrast, the evidence indicates no systematic differences between never smokers and current smokers. Taken together, these results are more consistent with health selection and reverse causality than with a protective causal effect of cessation. Chronic disease diagnosis, symptom onset, or clinical advice may induce smoking cessation, yielding the cross-sectional pattern in which former smokers appear to be at higher risk. Moreover, the current-smoker group may be shaped by selective survival and health-based selection, potentially attenuating estimates of the net protective effect of quitting. Moreover, our evidence suggests that, in a cross-sectional framework, smoking cessation is better conceptualized as a behavioral response embedded in the disease process rather than as a purely proactive investment in health improvement. Accordingly, the smoking-status indicator likely reflects not only the health consequences of prior smoking exposure, but also social processes tied to healthcare seeking, diagnosis, and subsequent behavioral adjustment. Empirically, future research could incorporate measures of cessation timing and years since quitting, along with prior smoking duration and intensity, to further decompose the observed ‘higher risk among former smokers’ into two mechanisms: cumulative exposure and disease-induced cessation. Where feasible, longitudinal data should be used to impose temporal ordering through lagged structures (e.g. prior dependence-subsequent onset), thereby reducing identification bias from reverse causality.

### Limitations

This study has the following limitations. First, the cross-sectional design does not permit firm temporal ordering among tobacco dependence, physical activity, depressive symptoms, and chronic disease. Tobacco dependence and depressive symptoms may be reciprocally related: depressive symptoms may increase smoking or dependence, consistent with self-medication dynamics, while tobacco dependence may also worsen psychological distress. Chronic disease may similarly shape both physical activity and depressive symptoms, as existing illness can reduce exercise participation and adversely affect mental health. Accordingly, the estimated path coefficients should be interpreted as model-based decompositions under a theoretically specified sequence rather than as definitive evidence of temporal mediation. Future research should exploit the longitudinal structure of CHARLS by using multiple panel waves to test whether baseline tobacco dependence predicts subsequent changes in physical activity and depressive symptoms and, in turn, later chronic disease onset. Longitudinal mediation or cross-lagged models based on repeated measures would provide a stronger basis for evaluating temporal ordering and potential reciprocal relationships. Second, tobacco dependence severity was approximated using daily cigarette consumption rather than the full FTND scale. Although smoking intensity is closely related to nicotine dependence, it does not capture other dimensions such as time to first cigarette, difficulty refraining from smoking, or smoking when ill. The findings should therefore be interpreted as evidence based on a consumption-based proxy rather than a validated multidimensional dependence measure. Third, self-report bias, particularly underreporting among women, may also attenuate estimates; unmeasured confounders such as pack-years remain possible. Additionally, the mediation analysis was restricted to current smokers; the findings, therefore, address only the pathways within the smoking population and do not generalize to former or never smokers.

## CONCLUSIONS

This secondary analysis of CHARLS 2020 data shows an overall positive gradient between a cigarette consumption-based proxy for tobacco dependence severity and chronic disease risk among older Chinese adults. Higher pre-cessation dependence is associated with persistent hypertension and dyslipidemia risk among former smokers. Descriptive subgroup variation appeared more pronounced among agricultural hukou holders, although formal interaction tests did not reach statistical significance.

The results suggest the value of moving beyond binary smoking classifications toward dependence-stratified approaches in screening and surveillance. Such considerations may inform tobacco control efforts under Healthy China 2030, particularly for rural populations. Longitudinal analyses leveraging the full CHARLS panel, along with biomarker data, would further clarify these associations. The framework may offer insights for other aging societies facing similar tobacco and NCD challenges.

## Supplementary Material



## Data Availability

The data supporting this research are available from the following source: https://charls.pku.edu.cn/en/

## PROVENANCE AND PEERE REVIEW

Not commissioned; externally peer reviewed.
