# Supplementary material for: Tobacco dependence severity, cessation legacy and serial mediation in aging China
Source: Tob Induc Dis. 2026 May 22;24:10.18332/tid/221187. doi: 10.18332/tid/221187 (PMC13197887; doi:10.18332/tid/221187)
Supplement: Supplementary file 1 [file TID-24-64-s1.pdf]

Appendix

Supplementary table 1. Definition, measurement and coding of key variables

Variable	CHARLS item(s)	Definition / measurement	Coding / values	Role in analysis
Current tobacco dependence	DA050_1	Consumption-based proxy for dependence severity among current smokers, based on average cigarettes per day at interview.	Mild (<10), moderate (10–20), severe (>20) cigarettes/day; approximation of dependence severity, not a full FTND measure.	Primary independent variable
Pre-cessation tobacco dependence	DA050_2	Consumption-based proxy for dependence severity among former smokers, based on average cigarettes per day before quitting.	Mild (<10), moderate (10–20), severe (>20) cigarettes/day; same thresholds as current dependence.	Primary independent variable
Current smoking status	DA047	Smoking status at interview.	1 = current smoker, 2 = former smoker, 3 = never smoker; entered as indicators with current smoker as the reference.	Supplementary predictor / grouping variable
Any chronic disease	DA003_1–DA003_15	Indicator of at least one physician-diagnosed chronic condition; used to capture overall morbidity burden.	1 = at least one condition, 0 = none; missing if all 15 disease items are missing.	Primary dependent variable
Chronic-condition count	DA003_1–DA003_15	Count of physician-diagnosed chronic conditions from the 15-item checklist.	Discrete count, 0–15; higher values indicate greater comorbidity burden.	Supplementary outcome

**Variable**	**CHARLS item(s)**	**Definition / measurement**	**Coding / values**	**Role in analysis**
Disease-specific chronic conditions	DA003_1–DA003_15	Binary indicators for each of the 15 physician-diagnosed chronic conditions retained for disease-specific analyses.	1 = yes, 0 = no; non-substantive responses treated as missing.	Supplementary outcomes
Physical exercise participation	DA033_*; DA037_*	Whether any reported activity is undertaken primarily for physical exercise.	1 if any DA037 item equals 3 ("for physical exercise"), 0 otherwise; missing if all DA037 items are missing.	Mediator
Depressive symptoms	DC016–DC025	Total depressive symptom score from the 10-item CHARLS depression module.	Continuous summed score: DC020 and DC023 reverse-coded so that higher values indicate more severe symptoms.	Mediator
Gender	+XRGender (from BA001, BA002)	Cross-wave harmonized measure of respondent sex/gender.	0 = male, 1 = female.	Covariate/ heterogeneity variable
Age	+XRAge (from prior birth-year data and BA003)	Age at interview, harmonized by CHARLS.	Continuous, in years.	Covariate

Marital status	BA011	Respondent’s marital status.	Categorical; collapsed in models according to the study specification.	Covariate
Hukou type	BA009	Household registration status.	1=agricultural, 2=non-agricultural/unified for heterogeneity analyses; “no hukou” treated as missing.	Covariate/ heterogeneity variable
Years of education	BA010; Zredu; xrtype	Harmonized 2020 educational attainment recoded into years of schooling.	Continuous; higher values indicate more years of schooling.	Covariate
Logged total income	GA001; GA002; GA005_s1–GA005_s10	Natural logarithm of annual wage/bonus income plus annual transfer income.	Continuous logged monetary measure.	Covariate

Current employment status	FA004; FA005	Whether the respondent is working or attached to a job at interview.	1 = employed / has a job; 0 = not employed; missing if status cannot be jointly determined.	Covariate

Notes:Tobacco dependence is measured as a consumption-based proxy rather than with the full FTND; non-substantive responses are treated as missing throughout. The 15 chronic conditions included in the questionnaire were: hypertension; dyslipidemia (hyperlipidemia or hypolipidemia); diabetes or elevated blood glucose (including impaired glucose tolerance and elevated fasting blood glucose); cancer or malignant tumor (excluding minor skin cancer); chronic lung disease, such as chronic bronchitis, emphysema, or cor pulmonale (excluding tumor or cancer); liver disease (excluding fatty liver, tumor, or cancer); heart disease (e.g., myocardial infarction, coronary heart disease, angina, congestive heart failure, and other heart diseases); stroke; kidney disease (excluding tumor or cancer); stomach or digestive disease (excluding tumor or cancer); emotional or psychiatric problems; memory-related disease (e.g., Alzheimer's disease and brain atrophy); Parkinson's disease; arthritis or rheumatism; and asthma (not classified as lung disease).

Supplementary Table 2. Logistic Regression Results of the Effects of Pre-Cessation Tobacco Dependence on the Risk of Specific Chronic Diseases, CHARLS 2020

VARIABLES	Model 1<br>Hypertensive AOR (N=1169)	Model 2<br>Hyperlipidemia or hypolipidemia AOR (N=1490)
Age	1.02(1.00-1.04)	1.02**(1.00-1.05)
Years of education	1.02(0.97- 1.07)	1.07**(1.01-1.12)
Logged Total income	0.94(0.82-1.09)	0.86**(0.75-0.98)
Pre-cessation level of tobacco dependence (Ref.=mild)		
Moderate	1.56**(1.02-2.37)	1.77***(1.17- 2.67)
Severe	1.81**(1.14-2.87)	1.89***(1.19-3.00)
Gender (Ref.=male)	1.00(0.46-2.15)	1.69*(0.91-3.17)
Marriage status (Ref.=Other	1.19(0.75-1.88)	1.15(0.76-1.75)
Hukou status		
Non-Agricultural HuKou	1.12(0.67-1.88)	1.32(0.79- 2.20)
Unified Residence HuKou	1.17(0.64-2.15)	1.14(0.61-2.12)
Work or not (Ref.=No)	1.24(0.79-1.94)	1.26(0.80-1.96)
Constant	0.04***(0.00- 0.36)	0.03***(0.00-0.26)

Notes: AOR means adjusted odds ratio, with 95% confidence intervals in brackets. **** p<0.001, *** p<0.01, ** p<0.05, * p<0.1

Supplementary Table 3. Logistic Regression Results of the Effects of Current Smoking Status on the Risk of Specific Chronic Diseases, CHARLS 2020

VARIABLES	Hypertension AOR (N=4042)	Hyperlipidemia /Hypolipidemia AOR (N=5025)	Diabetes/ Elevated Blood Glucose AOR (N=5508)	Chronic Lung Disease AOR (N=5335)	Heart Disease AOR (N=5208)	Stroke Disease AOR (N=5747)
Quit	1.29**	1.27**	1.40***	1.37**	1.68****	1.59**
	(1.04-1.59)	(1.04-1.57)	(1.09- 1.82)	(1.07- 1.75)	(1.32-2.15)	(1.10-2.30)
Never Smoked	1.17	1.27	1.41	1.07	1.21	1.54
	(0.79- 1.74)	(0.90-1.81)	(0.91-2.20)	(0.69-1.68)	(0.77- 1.92)	(0.81-2.90)
Control Var.	yes	yes	yes	yes	yes	yes
Constant	0.08****	0.05****	0.03****	0.01****	0.03****	0.05***
	(0.02-0.26)	(0.02-0.16)	(0.01-0.14)	(0.00-.04)	(0.01-0.12)	(0.01-0.37)

Notes: AOR means adjusted odds ratio, with 95% confidence intervals in brackets. **** p<0.001, *** p<0.01, ** p<0.05, * p<0.1.

Supplementary Table 4. Interaction Models of Tobacco Dependence with Hukou Status and Gender, CHARLS 2020, (N = 3637)

VARIABLES	Current level of tobacco dependence× Gender<br>AOR	Current level of tobacco dependence× Hukou<br>AOR
Current level of tobacco dependence (Ref.=mild)		
Moderate	1.17* (1.00–1.37)	1.26**(1.05–1.51)
Severe	1.21* (0.97–1.51)	1.24*(0.96–1.61)
Gender (Ref=male)	1.09 (0.78–1.53)	
CTD # Gender		
Moderate #Female	1.59 (0.91–2.78)	
Severe # Female	1.00 (0.26–3.78)	
Hukou (Ref.=Agri.)		1.31**(1.00–1.70)
Moderate # non-Agri.		0.87 (0.63–1.21)
Severe # non-Agri.		0.98 (0.60–1.60)
Control Variables	Yes	Yes
Constant	0.17 ****(0.07- 0.40)	0.16****(0.07- 0.39)

Notes: AOR means adjusted odds ratio, with 95% confidence intervals in brackets. **** p<0.001, *** p<0.01, ** p<0.05, * p<0.1.

Supplementary Table 5. Logistic Regression Results of the Subsample Analyses, CHARLS 2020

VARIABLES	Male<br>Chronic Disease AOR (N=3368)	Female<br>Chronic Disease AOR	Agricultural<br>Chronic Disease AOR	Non-Agri.& Unified<br>Chronic Disease AOR
Age	1.01***(1.00- 1.02)	1.01(0.98- 1.04)	1.01*(1.00- 1.02)	1.02**(1.00- 1.04)
Years of education	1.01(0.99-1.03)	1.06(0.97-1.16)	1.01(0.98-1.03)	1.03(0.99-1.07)
Logged Total income	1.02(0.96-1.08)	1.15(0.92-1.44)	1.03(0.96-1.10)	1.04(0.93-1.18)
Current level of tobacco dependence (Ref.=mild)				
Moderate	1.17**(1.00-1.38)	2.06**(1.19-0.58)	1.28***(1.07-1.54)	1.09(0.82-1.44)
Severe	1.22*(0.97-1.53)	1.30(0.34-4.92)	1.27*(0.98-1.64)	1.17(0.77- 1.80)
Marriage status	0.93(0.78-1.11)	0.67(0.39-1.16)	0.91(0.76-1.10)	0.90(0.64-1.27)
Hukou status (Ref.=Agricultural)				
Non-Agricultural HuKou	1.37***(1.10-1.71)	0.71(0.28- 1.79)		
Unified Residence HuKou	1.11(0.87-1.43)	0.48(0.18-1.25)		
Work or not (Ref.=No)	0.77***(0.64-0.94)	0.87(0.35-2.15)	0.69***(0.55-0.87)	1.00(0.71-1.41)
Gender (Ref.=male)			1.32*(0.97-1.81)	1.10(0.64-1.89)
Constant	0.17****(0.07-0.44)	0.10(0.01-1.89)	0.21***(0.07-0.62)	0.08***(0.01-0.44)

Notes: AOR means adjusted odds ratio, with 95% confidence intervals in brackets. **** p<0.001, *** p<0.01, ** p<0.05, * p<0.1.

Supplementary Table 6. Correlation matrix of the key variables, CHARLS 2020 (N=3637)

Variables	1	2	3	4	5	6	7	8	9	10	11	12
1.Current	1.0000											
2.Chronic	0.0108	1.0000										
3.physical	-0.1076**	0.0407**	1.0000									
4.Depressio	-0.0426**	0.1341**	-0.0290**	1.0000								
5.Gender	-0.1418**	0.0189**	0.0141	0.1753**	1.0000							
6.Age	-0.1600**	0.0987**	0.1650**	0.0808**	-0.0348**	1.0000						
7.Marital	-0.0323*	0.0317**	0.0207**	0.1224**	0.1070**	0.1768**	1.0000					
8.Years of	0.0581**	-0.0129	0.0916**	-0.2227**	-0.2877**	-0.2544**	-0.1227**	1.0000				
9.Non-	-0.0313*	0.0272**	0.1510**	-0.1095**	-0.0490**	0.0191**	-0.0422**	0.3318**	1.0000			
10. Unified	-0.0043	0.0145*	0.0833**	-0.0719**	0.0030	0.0344**	-0.0001	0.1322**	-0.1440**	1.0000		
11.Logged	0.1076**	-0.0346**	0.0429**	-0.2786**	-0.1968**	-0.3196**	-0.0813**	0.5131**	0.4036**	0.2272**	1.0000	
12.Work	0.1529**	-0.0733**	-0.1494**	-0.1515**	-0.2024**	-0.4134**	-0.0438**	0.2407**	0.0214**	-0.0089	0.3981**	1.000

Notes: Hukou status is measured as a three-category variable: agricultural, non-agricultural, and unified hukou. In the correlation analysis, hukou is represented by a set of dichotomous indicators to avoid imposing an artificial ordinal or metric structure. Agricultural hukou is treated as the reference category, and two dummy variables are included for non-agricultural hukou (**hukou_2** = 1) and unified hukou (**hukou_3** = 1), with 0 otherwise. All correlations and significance tests are based on the complete-case sample (listwise deletion). Coefficients involving these dummy variables should therefore be interpreted as associations between hukou category membership and other variables, rather than as distances between hukou categories. ****P <.001，**p <.01，*p <.05*

© 2026 Li W. et al.
